# 

**Published:** 1859-08

**Authors:** 


### CONTENTS.

Dental Education. By J. TAFT .... 53
Alveolar Hemorrhage. By GEO. WATT .... 57
A Squib .... 61
Treatment of Recently Exposed Nerves with Nitric Acid—with Cases. By J. TAFT .... 63
Cheap Dentistry and its Tendencies. By PROF. JOHN R. ALLEN, N. Y. .... 65
Gold—No. VI. By PROF. AUSTIN .... 66
Death from Chloroform .... 72
The Lucifer Match Disease, or Rotting of the Jaw Bone .... 74
A Few Ideas on Mechanical Dentistry. By F. J. S. GORGAS, D. D. S. .... 77
Dr. Lankester on Food .... 79
Artificial Production of Bone .... 80
Neuralgia Faciei .... 81
Fracture of the Lower Jaw .... 83
Poisoning by Chloroform .... 83
Hemorrhage after the Extraction of Three Molar Teeth .... 86
Stomatitis Materni. By DAVID PRINCE, M. D. .... 87
Plastic Filling .... 89
Recent Dental Patents .... 91
Correspondence .... 91
Editorial .... 95

